# Supplementary material for: Doxazosin for the treatment of mental health disorders: A scoping review
Source: PLOS Ment Health. 2025 Nov 19;2(11):e0000494. doi: 10.1371/journal.pmen.0000494 (PMC12798304; doi:10.1371/journal.pmen.0000494)
Supplement: S1 Data — (PDF) [file pmen.0000494.s002.pdf]

ClinicalTrials.gov

NIH National Library of Medicine
National Center for Biotechnology Information

**The U.S. government does not review or approve the safety and science of all studies listed on this website.**

Read our full disclaimer (https://clinicaltrials.gov/about-site/disclaimer) for details.

Completed

## Pharmacogenetic Trial of Doxazosin for Treatment of Cocaine Abuse

**ClinicalTrials.gov ID** NCT01953432

**Sponsor** VA Office of Research and Development

**Information provided by** VA Office of Research and Development (Responsible Party)

**Last Update Posted** 2020-02-20

# Study Details Tab

## Study Overview

### Brief Summary

Cocaine use disorders affect approximately 1.5 million Americans annually. Currently, there are no US Food and Drug Administration approved medications for treatment of cocaine dependence; however, both animal and human studies suggest that medications affecting the noradrenergic system can reduce cocaine craving and use. The investigators will study the effect of doxazosin, an alpha-1 adrenergic

Feedback

antagonist, in reducing cocaine use and anxiety symptoms among cocaine-dependent individuals. In addition, the investigators will identify genetic subpopulations of participants who preferentially respond to the medication.

**Detailed Description**

The noradrenergic system, especially the alpha 1-adrenergic receptor, may play an important role in cocaine addiction in humans. Doxazosin is a long-acting and selective alpha 1-adrenergic receptor blocker, which inhibits the binding of norepinephrine to alpha receptors in the autonomic nervous system. This study will evaluate the efficacy of doxazosin in reducing cocaine-using behavior in treatment seeking cocaine-dependent individuals, and will guide future pharmacotherapy trials using Doxazosin or related alpha 1 receptor antagonists for treatment of cocaine addiction.

This 12-week double-blind, placebo controlled clinical trial will provide treatment for cocaine-dependent patients and includes a 8-week maintenance medication trial (weeks 3-10). At the end of this period, subjects will have a 2-week study medication taper (weeks 11-12). Qualifying subjects will be randomized to receive Doxazosin up to 8 mg/day or placebo.

At the beginning of week 1, participants will receive Doxazosin, or placebo according to their randomized assignments, and are maintained on these agents through week 12. At the end of the study (weeks 11-12), participants will undergo discontinuation from active/inactive medication over a 2-week period. Subjects who wish to be transferred to an appropriate treatment program or research program will be referred to upon request, during the study weeks 11-12.

**Official Title**

Pharmacogenetic Trial of Noradrenergic Medication for Treatment of Cocaine Abuse

**Conditions**

Cocaine Dependence

**Intervention / Treatment**

- Drug: Doxazosin
- Drug: Placebo

**Other Study ID Numbers**

- CLIN-014-12F

- 1IK2CX000946-01 ( U.S. NIH Grant/Contract ) (https://reporter.nih.gov/quickSearch/1IK2CX000946-01)

**Study Start (Actual)**

2014-04-01

**Primary Completion (Actual)**

2017-09-01

**Study Completion (Actual)**

2017-10-01

**Enrollment (Actual)**

43

**Study Type**

Interventional

**Phase**

Phase 2

**Resource links provided by the National Library of Medicine**

MedlinePlus (https://medlineplus.gov/) related topics: Cocaine (https://medlineplus.gov/cocaine.html) Drug Safety (https://medlineplus.gov/drugsafety.html) Drug Use and Addiction (https://medlineplus.gov/druguseandaddiction.html) Drugs and Young People (https://medlineplus.gov/drugsandyoungpeople.html) Pregnancy and Substance Use (https://medlineplus.gov/pregnancyandsubstanceuse.html)

Drug Information (https://dailymed.nlm.nih.gov/dailymed/) available for: Doxazosin (https://dailymed.nlm.nih.gov/dailymed/search.cfm?labeltype=human&query=Doxazosin)

FDA Drug and Device Resources (https://clinicaltrials.gov/fda-links)

## Contacts and Locations

This section provides contact details for people who can answer questions about joining this study, and information on where this study is taking place.

To learn more, please see the Contacts and Locations section in How to Read a Study Record (https://clinicaltrials.gov/study-basics/how-to-read-study-record#contacts-and-locations).

This study has 1 location

### United States

#### Texas Locations

**Houston, Texas, United States, 77030**
Michael E. DeBakey VA Medical Center, Houston, TX

## Participation Criteria

Researchers look for people who fit a certain description, called eligibility criteria. Some examples of these criteria are a person's general health condition or prior treatments.

For general information about clinical research, read Learn About Studies (https://clinicaltrials.gov/study-basics/learn-about-studies).

## Eligibility Criteria

### Description

Inclusion Criteria:

1. Signed informed consent form and HIPAA authorization form
2. Subject is cooperative, understands the risks and benefits, and is willing and able to adhere to study requirements
3. Any race or ethnic origin
4. Diagnosis of cocaine-dependence according to DSM-IV criteria
5. Between the ages of 18 and 64
6. Must be current users of cocaine with self-reported use of cocaine within the last 90 days, or at least one cocaine-positive urine during screening.
7. Women of childbearing age are eligible to be included in the study if they have a negative pregnancy test at screening, agree to adequate contraception to prevent pregnancy, to have monthly pregnancy tests, and they understand the risk of fetal toxicity due to medication.
8. Must be in good general health as determined by self-report and/or CPRS-based medical history, general clinical examination conducted by a study physician, and lab tests. HIV testing will be recommended but is not required for participation in this study.
9. Motivated to discontinue or reduce cocaine use during the period of the study, as evidenced both by the judgment of the Investigator or designee and by the subject's compliance level with the requirement for attendance at clinic visits, such that weekly urine sample requirements for inclusion criteria are fully met.

Exclusion Criteria:

1. Current diagnosis of other drug dependence, especially alcohol or benzodiazepine dependence, or abuse (other than cocaine, tobacco, or cannabis)
2. Significant medical conditions (e.g., major cardiovascular, renal, endocrine, hepatic disorders) such as abnormal liver function (with laboratory findings of SGOT or SGPT greater than three times normal), hypotension, a current cardiac condition that in the opinion of the investigator would contraindicate Doxasozin treatment, and those having a high risk of cardiovascular disease, seizure disorders, or another significant underlying medical condition which would contraindicate Doxazosin treatment
3. Lifetime schizophrenia, bipolar disorder, or other psychotic disorders (excluding substance-induced psychotic disorders)
4. Actively considering plans of suicidality or homicidality

5. Women planning to become pregnant or breastfeed during the study, refusal to use a reliable form of birth control, or refusal of monthly pregnancy testing
6. Subjects who are prescribed certain anti-hypertension drugs (i.e. doxasozin) will be excluded because these medications may interact with Doxazosin's brain effects in reducing cocaine abuse
7. Subject has participated in another clinical trial or received any other investigational compound within 7 days prior to being randomized into this study

**Ages Eligible for Study**

18 Years to 64 Years (Adult )

**Sexes Eligible for Study**

All

**Accepts Healthy Volunteers**

Yes

## Study Plan

This section provides details of the study plan, including how the study is designed and what the study is measuring.

### How is the study designed?

## Design Details

**Primary Purpose** : Treatment
**Allocation** : Randomized
**Interventional Model** : Parallel Assignment
**Masking** : Quadruple (Participant, Care Provider, Investigator, Outcomes Assessor)

### Arms and Interventions

| Participant Group/Arm | Intervention/Treatment |
|---|---|
| Experimental: Doxazosin<br><br>Doxazosin is a long-acting and selective alpha 1-NE blocker, which inhibits the binding of norepinephrine to alpha receptors in the autonomic nervous system. | Drug: Doxazosin<br><br>• Doxazosin is initiated at 2 mg/wk, and titrated up to a maximum of 8 mg/day over approximately 4 weeks. Participants will be maintained on 8mg daily dosing until week 13. The subjects will undergo the discontinuation from the study medication during weeks 14 -15.<br>• Other Names:<br>  ◦ Cardura (Doxazosin Mesylate) |
| Placebo Comparator: Placebo<br><br>Matched placebo daily dosing. | Drug: Placebo<br><br>• Matched placebo daily dosing<br>• Other Names:<br>  ◦ Sugar pills (capsule) |

## What is the study measuring?

**Primary Outcome Measures**

| Outcome Measure | Measure Description | Time Frame |
|---|---|---|
| Percentage of Cocaine-positive Urines | Over period of 12 weeks with 43 participants total (Doxazosin group = 22; Placebo group = 21), the overall percentage of cocaine positive urines per treatment group | Up to 12 weeks, or for the duration of the participant's involvement in the study |

## Collaborators and Investigators

This is where you will find people and organizations involved with this study.

**Sponsor**

### VA Office of Research and Development

**Collaborators**

- Baylor College of Medicine

**Investigators**

- Principal Investigator: Daryl I Shorter, MD, Michael E. DeBakey VA Medical Center, Houston, TX

## Study Record Dates

These dates track the progress of study record and summary results submissions to ClinicalTrials.gov. Study records and reported results are reviewed by the National Library of Medicine (NLM) to make sure they meet specific quality control standards before being posted on the public website.

### Study Registration Dates

**First Submitted**
2013-09-10

**First Submitted that Met QC Criteria**
2013-09-24

**First Posted (Estimated)**
2013-10-01

### Results Reporting Dates

**Results First Submitted**
2019-11-12

**Results First Submitted that Met QC Criteria**
2020-02-14

**Results First Posted**
2020-02-20

### Study Record Updates

**Last Update Submitted that met QC Criteria**

2020-02-14

**Last Update Posted**

2020-02-20

**Last Verified**

2020-02

## More Information

### Terms related to this study

**Keywords Provided by VA Office of Research and Development**

Cocaine-Related Disorders
Cocaine
Doxazosin
Cardiovascular Agents
Antihypertensive Agents
Adrenergic alpha-1 Receptor Antagonists
Adrenergic alpha-Antagonists
Adrenergic Antagonists
Molecular Mechanisms of Pharmacological Action
Neurotransmitter Agents
Therapeutic Uses
Pharmacologic Actions
Substance-Related Disorders

**Additional Relevant MeSH Terms**

Chemically-Induced Disorders
Mental Disorders
Cocaine-Related Disorders
Substance-Related Disorders
Prazosin
Quinazolines
Heterocyclic Compounds, 2-Ring
Heterocyclic Compounds, Fused-Ring
Heterocyclic Compounds
Substandard Drugs
Pharmaceutical Preparations
Dosage Forms
Doxazosin
Counterfeit Drugs
Capsules

HHS Vulnerability Disclosure

## Plan for Individual Participant Data (IPD)

**Plan to Share Individual Participant Data (IPD)?**

No

## Drug and device information, study documents, and helpful links

**Studies a U.S. FDA-Regulated Drug Product**

Yes

**Studies a U.S. FDA-Regulated Device Product**

No

**Product Manufactured in and Exported from the U.S.**

Yes

**Study Documents Provided by VA Office of Research and Development**

- Study Protocol and Statistical Analysis Plan (https://cdn.clinicaltrials.gov/large-docs/32/NCT01953432/Prot_SAP_000.pdf) [PDF, 0.09MB, 2019-03-14]

ClinicalTrials.gov

NIH National Library of Medicine
National Center for Biotechnology Information

**The U.S. government does not review or approve the safety and science of all studies listed on this website.**

Read our full disclaimer (https://clinicaltrials.gov/about-site/disclaimer) for details.

Completed

## Pharmacogenetic Trial of Doxazosin for Treatment of Cocaine Abuse

**ClinicalTrials.gov ID** NCT01953432

**Sponsor** VA Office of Research and Development

**Information provided by** VA Office of Research and Development (Responsible Party)

**Last Update Posted** 2020-02-20

# Results Posted Tab

## Results Overview

**Conditions**

Cocaine Dependence

**Intervention/Treatment**

Feedback

- Drug: Doxazosin
- Drug: Placebo

### Other Study ID Numbers

- CLIN-014-12F
- 1IK2CX000946-01 ( U.S. NIH Grant/Contract ) (https://reporter.nih.gov/quickSearch/1IK2CX000946-01)

### Study Design

**Allocation**: Randomized

**Interventional Model**: Parallel Assignment

**Masking**: Quadruple (Participant, Care Provider, Investigator, Outcomes Assessor)

**Primary Purpose**: Treatment

### Results Point of Contact

**Name/Title:** Daryl Shorter, MD
**Organization:** Michael E. DeBakey VA Medical Center
**Phone:** 713-798-4870
**Email:** shorter@bcm.edu

### Enrollment (Actual)

43

### Study Type

Interventional

## Study Record Dates

These dates track the progress of study record and summary results submissions to ClinicalTrials.gov. Study records and reported results are reviewed by the National Library of Medicine (NLM) to make sure they meet specific quality control standards before being posted on the public website.

### Study Registration Dates

**First Submitted**

2013-09-10

**First Posted (Estimated)**

2013-10-01

### Results Reporting Dates

**Results First Submitted**

2019-11-12

**Results First Posted**

2020-02-20

### Study Record Updates

**Last Update Posted**

2020-02-20

**Last Verified**

2020-02

## Participant Flow

**Recruitment Details**

[Not Specified]

**Pre-assignment Details**

[Not Specified]

| Arm/Group Title | Doxazosin | Placebo |
|---|---|---|
| Arm/Group Description | Doxazosin is a long-acting and selective alpha 1-NE blocker, which inhibits the binding of norepinephrine to alpha receptors in the autonomic nervous system.<br><br>Doxazosin (target 8mg/day) Induction - Week 1: 1mg once daily over days 1--3; 2mg once daily over days 4-7; Week 2: 4mg once daily over days 8-10; 8mg once daily over days 11-14; Week 3: 8mg once daily over days 15-end of week 10<br><br>Doxazosin tapered over weeks 11-12 -- Week 11: 4mg on Monday, Tuesday, Wednesday, and Thursday and 1mg for the duration of week 11. During week 12, subjects will receive 1mg on Monday, Tuesday, and Wednesday only. No further medication will be given for the remainder of week 12. | Matched placebo daily dosing.<br><br>Placebo: Matched placebo daily dosing |

Period Title: **Overall Study**

| | | |
|---|---|---|
| Started | 22 | 21 |

| | | |
|---|---|---|
| Completed | 8 | 6 |
| Not Completed | 14 | 15 |

## Baseline Characteristics

| Arm/Group Title | Doxazosin | Placebo | Total |
|---|---|---|---|
| Arm/Group Description | Doxazosin is a long-acting and selective alpha 1-NE blocker, which inhibits the binding of norepinephrine to alpha receptors in the autonomic nervous system.<br><br>Doxazosin (target 8mg/day) Induction - Week 1: 1mg once daily over days 1--3; 2mg once daily over days 4-7; Week 2: 4mg once daily over days 8-10; 8mg once daily over days 11-14; Week 3: 8mg once daily over days 15-end of week 10<br><br>Doxazosin tapered over weeks 11-12 -- Week 11: 4mg on Monday, Tuesday, Wednesday, and Thursday and 1mg for the duration of week 11. During week 12, subjects will receive 1mg on Monday, Tuesday, and Wednesday only. No further medication will be given for the remainder of week 12. | Matched placebo daily dosing.<br><br>Placebo: Matched placebo daily dosing | Total of all reporting groups |
| Overall Number of Baseline Participants | 22 | 21 | 43 |
| Baseline Analysis Population Description | [Not Specified] | | |

Expand all / Collapse all

Age, Categorical

Measure Type: Count of Participants | Unit of measure: Participants

| Number Analyzed | 22 participants | 21 participants | 43 participants |
|---|---|---|---|
| <=18 years | 0 0.0% | 0 0.0% | 0 0.0% |
| Between 18 and 65 years | 22 100.0% | 21 100.0% | 43 100.0% |
| >=65 years | 0 0.0% | 0 0.0% | 0 0.0% |

**Age, Continuous**

Mean (Full Range) | Unit of measure: years

| Number Analyzed | 22 participants | 21 participants | 43 participants |
|---|---|---|---|
| | 53.4 (26 to 61) | 54.9 (43 to 62) | 54.2 (26 to 62) |

**Sex: Female, Male**

Measure Type: Count of Participants | Unit of measure: Participants

| Number Analyzed | 22 participants | 21 participants | 43 participants |
|---|---|---|---|
| Female | 2 9.1% | 0 0.0% | 2 4.7% |
| Male | 20 90.9% | 21 100.0% | 41 95.3% |

**Race (NIH/OMB)**

Measure Type: Count of Participants | Unit of measure: Participants

| | | | |
|---|---|---|---|
| Number Analyzed | 22 participants | 21 participants | 43 participants |
| American Indian or Alaska Native | 0 0.0% | 1 4.8% | 1 2.3% |
| Asian | 0 0.0% | 0 0.0% | 0 0.0% |
| Native Hawaiian or Other Pacific Islander | 0 0.0% | 0 0.0% | 0 0.0% |
| Black or African American | 19 86.4% | 17 81.0% | 36 83.7% |
| White | 3 13.6% | 3 14.3% | 6 14.0% |
| More than one race | 0 0.0% | 0 0.0% | 0 0.0% |
| Unknown or Not Reported | 0 0.0% | 0 0.0% | 0 0.0% |

**Region of Enrollment**

Measure Type: Count of Participants | Unit of measure: Participants

| | | | |
|---|---|---|---|
| Number Analyzed | 22 participants | 21 participants | 43 participants |
| United States | 22 100.0% | 21 100.0% | 43 100.0% |

**Lifetime cocaine years**

Mean (Full Range) | Unit of measure: years

| | | | |
|---|---|---|---|
| Number Analyzed | 22 participants | 21 participants | 43 participants |

| | | | |
|---|---|---|---|
| | 24.5<br>(4 to 43) | 24.1<br>(14 to 38) | 24.3<br>(4 to 43) |

## Outcome Measures

1. Percentage of Cocaine-positive Urines

Type: Primary | Time Frame: Up to 12 weeks, or for the duration of the participant's involvement in the study

| | |
|---|---|
| Description | Over period of 12 weeks with 43 participants total (Doxazosin group = 22; Placebo group = 21), the overall percentage of cocaine positive urines per treatment group |
| Time Frame | Up to 12 weeks, or for the duration of the participant's involvement in the study |
| Analysis Population Description | Study population was comprised of 43 cocaine-dependent individuals who met inclusion criteria for this study. |

| Arm/Group Title | Doxazosin | Placebo |
|---|---|---|
| Arm/Group Description | Doxazosin is a long-acting and selective alpha 1-NE blocker, which inhibits the binding of norepinephrine to alpha receptors in the autonomic nervous system.<br><br>Doxazosin (target 8mg/day) Induction - Week 1: 1mg once daily over days 1--3; 2mg once daily over days 4-7; Week 2: 4mg once daily over days 8-10; 8mg once daily over days 11-14; Week 3: 8mg once daily over days 15-end of week 10<br><br>Doxazosin tapered over weeks 11-12 -- Week 11: 4mg on Monday, Tuesday, Wednesday, and Thursday and 1mg for the duration of week 11. During week 12, subjects will receive 1mg on Monday, Tuesday, and Wednesday only. No further medication will be given for the remainder of week 12. | Matched placebo daily dosing.<br><br>Placebo: Matched placebo daily dosing |
| Overall Number of Participants Analyzed | 22 | 21 |
| Measure Type: Number \| Unit of Measure: percentage of cocaine-positive urines | 67.6 | 69 |

Expand all / Collapse all

Statistical Analysis 1

Statistical Analysis Overview

| | |
|---|---|
| Comparison Group Selection | Doxazosin |
| Comments | [Not Specified] |
| Type of Statistical Test | Other |
| Comments | [Not Specified] |

Method of Estimation

| | |
|---|---|
| Estimation Parameter | Odds Ratio (OR) |
| Estimated Value | 0.75 |
| Estimation Comments | Probability of 0.75 that the OR exceeded 1.00 (OR = 1.01, 95% CI = 0.98-1.05) |

Statistical Analysis 2

Statistical Analysis Overview

| | |
|---|---|
| Comparison Group Selection | Placebo |
| Comments | [Not Specified] |
| Type of Statistical Test | Other |
| Comments | [Not Specified] |

| Method of Estimation | |
|---|---|
| Estimation Parameter | Odds Ratio (OR) |
| Estimated Value | 0.96 |
| Estimation Comments | Probability of 0.96 that the odds ratio exceeded 1.00 (OR = 1.03, 95% CI = 1.00-1.07) |

## Adverse Events

**Time Frame**

Adverse events were assessed at each visit (Monday, Wednesday, Friday) during the active (medication) portion of the study for each participant (12 weeks).

**Adverse Event Reporting Description**

[Not Specified]

| Arm/Group Title | Doxazosin | Placebo |
|---|---|---|
| Arm/Group Description | Doxazosin is a long-acting and selective alpha 1-NE blocker, which inhibits the binding of norepinephrine to alpha receptors in the autonomic nervous system.<br><br>Doxazosin: Doxazosin is initiated at 2 mg/wk, and titrated up to a maximum of 8 mg/day over approximately 4 weeks. Participants will be maintained on 8mg daily dosing until week 13. The subjects will undergo the discontinuation from the study medication during weeks 14 -15. | Matched placebo daily dosing.<br><br>Placebo: Matched placebo daily dosing |

Expand

## All-Cause Mortality

| Arm/Group Title | Doxazosin | Placebo |
|---|---|---|
| | Affected / at Risk (%) | Affected / at Risk (%) |
| Total | 0/22 (0.00%) | 0/21 (0.00%) |

## Serious Adverse Events

| Arm/Group Title | Doxazosin | | Placebo | |
|---|---|---|---|---|
| | Affected / at Risk (%) | # Events | Affected / at Risk (%) | # Events |
| Total | 0/22 (0.00%) | | 0/21 (0.00%) | |

## Other (Not Including Serious) Adverse Events

| Frequency Threshold for Reporting Other Adverse Events | 0% | | | |
|---|---|---|---|---|
| Arm/Group Title | Doxazosin | | Placebo | |
| | Affected / at Risk (%) | # Events | Affected / at Risk (%) | # Events |
| Total | 8/22 (36.36%) | | 5/21 (23.81%) | |
| **Gastrointestinal disorders** | | | | |
| Nausea * | 2/22 (9.09%) | 2 | 1/21 (4.76%) | 1 |
| Diarrhea * | 0/22 (0.00%) | 0 | 2/21 (9.52%) | 2 |
| Stomach Pain/Indigestion * | 1/22 (4.55%) | 1 | 1/21 (4.76%) | 1 |
| Constipation * | 1/22 (4.55%) | 1 | 0/21 (0.00%) | 0 |
| **General disorders** | | | | |
| Dry mouth * | 1/22 (4.55%) | 1 | 1/21 (4.76%) | 1 |
| Lightheadedness * | 1/22 (4.55%) | 1 | 1/21 (4.76%) | 1 |
| Dizziness * | 1/22 (4.55%) | 1 | 0/21 (0.00%) | 0 |
| Muscle twitching * | 0/22 (0.00%) | 0 | 1/21 (4.76%) | 1 |
| **Immune system disorders** | | | | |
| Swelling * | 0/22 (0.00%) | 0 | 1/21 (4.76%) | 1 |

| | | | | |
|---|---|---|---|---|
| Allergies * | 1/22 (4.55%) | 1 | 0/21 (0.00%) | 0 |
| **Infections and infestations** | | | | |
| Cold/Viral Illness * | 1/22 (4.55%) | 1 | 1/21 (4.76%) | 1 |
| **Musculoskeletal and connective tissue disorders** | | | | |
| Back Pain * | 0/22 (0.00%) | 0 | 1/21 (4.76%) | 1 |
| **Nervous system disorders** | | | | |
| Numbness * | 1/22 (4.55%) | 1 | 0/21 (0.00%) | 0 |
| **Psychiatric disorders** | | | | |
| Increased Drug Craving * | 0/22 (0.00%) | 0 | 1/21 (4.76%) | 1 |
| **Reproductive system and breast disorders** | | | | |
| Erectile Dysfunction * | 0/22 (0.00%) | 0 | 1/21 (4.76%) | 1 |
| **Respiratory, thoracic and mediastinal disorders** | | | | |
| Bronchitis * | 0/22 (0.00%) | 0 | 1/21 (4.76%) | 1 |

* Indicates events were collected by non-systematic assessment

HHS Vulnerability Disclosure

## Limitations and Caveats

There was a protocol amendment and the primary outcome measure was changed. The planned analysis was revised to a Bayesian approach to ensure more robust analysis of data given smaller sample size.

## Collaborators and Investigators

This is where you will find people and organizations involved with this study.

**Sponsor**

### VA Office of Research and Development

**Collaborators**

- Baylor College of Medicine

**Investigators**

- Principal Investigator: Daryl I Shorter, MD, Michael E. DeBakey VA Medical Center, Houston, TX

## More Information

Record History

**Certain Agreements**

Principal Investigators ARE employed by the organization sponsoring the study.
