# Supplementary material for: Doxazosin for the treatment of mental health disorders: A scoping review
Source: PLOS Ment Health. 2025 Nov 19;2(11):e0000494. doi: 10.1371/journal.pmen.0000494 (PMC12798304; doi:10.1371/journal.pmen.0000494)
Supplement: S2 Data — (PDF) [file pmen.0000494.s003.pdf]

ClinicalTrials.gov

NIH National Library of Medicine
National Center for Biotechnology Information

The U.S. government does not review or approve the safety and science of all studies listed on this website.

Read our full disclaimer (https://clinicaltrials.gov/about-site/disclaimer) for details.

Completed

Testing Doxazosin to Treat Stress Mechanisms in Alcoholism

ClinicalTrials.gov ID NCT02989493

Sponsor University of Wisconsin, Madison

Information provided by University of Wisconsin, Madison (Responsible Party)

Last Update Posted 2021-03-18

Study Details Tab

Study Overview

Brief Summary

Double-blind, placebo controlled, randomized controlled trial (RCT) for Alcohol Use Disorder examining the effects of doxazosin, a norepinephrine alpha1 receptor antagonist, on stress reactivity and clinical outcomes.

Detailed Description

Feedback

OBJECTIVES:

1. To translate the preclinical evidence from animal models to stress-induced relapse in humans via direct pharmacological antagonism of the noradrenergic system in abstinent alcoholics with doxazosin, an alpha1 noradrenergic receptor blocker.
2. To screen the efficacy of doxazosin to target stress-related relapse mechanisms in abstinent alcoholics as a cost-effective first step to repurpose this alpha1 noradrenergic antagonist for relapse prevention in addiction.

PARTICIPANTS:

136 participants with an Alcohol Use Disorder in early abstinence.

STUDY OVERVIEW:

136 adults with an Alcohol Use Disorder in early abstinence (1-8 weeks abstinent) will participate in a randomized controlled trial (RCT) to examine the efficacy of 8 mg doxazosin (vs. placebo, between-subjects) on stress reactivity and clinical outcome measures (e.g., drinks/week, alcohol craving) during a 8 week treatment period. Doxazosin's impact on stress-related relapse mechanisms will be assessed using a well-validated human model of stressor reactivity (No Shock, Predictable Shock, Unpredictable Shock [NPU] task) at baseline (pre-treatment) and after 4 weeks of treatment. The NPU task has strong translational ties to both methods (e.g., unpredictable vs. predictable electric shock) and measures (e.g., startle potentiation) from the preclinical literature in animals. This laboratory stress task serves as an attractive early surrogate endpoint post-treatment to assess treatment efficacy and examine stress mechanisms.

AIMS and HYPOTHESIS:

AIM 1: Examine effects of a therapeutic dose of doxazosin on responses to unpredictable stressors in NPU task. The aim is to obtain preliminary evidence via a laboratory surrogate endpoint to repurpose doxazosin for the treatment of stress-induced relapse mechanisms in alcoholism.

PREDICTIONS: Following four weeks of therapeutic dosing, doxazosin (8 mg vs. placebo, between-subjects) will selectively reduce response to unpredictable (vs. predictable) stressors indexed by physiological defensive reactivity (startle potentiation) and self-reported negative affect and craving in abstinent alcoholics.

AIM 2: Examine effects of a therapeutic dose of doxazosin on early clinical outcome measures. The aim is to obtain additional evidence via clinical outcome measures to repurpose doxazosin for the treatment of stress-induced relapse mechanisms in alcoholism.

PREDICTIONS: Following eight weeks of therapeutic dosing, doxazosin (8 mg vs. placebo, between-subjects) will increase continuous abstinence and decrease drinking days per week and drinks per week during the medication treatment period. Doxazosin will also decrease

craving measured during the 8th week of medication use when participants have achieved the maximum dose for 4.5 weeks.

AIM 3: Examine predictive validity of pre-treatment laboratory tests of noradrenergic relevant stress-reactivity on surrogate endpoint and clinical outcome measures. The aim is to link individual differences in stress reactivity at baseline (i.e. pre-treatment) to laboratory surrogate endpoints and early clinical outcome measures following therapeutic dosing.

PREDICTIONS: Higher pre-treatment reactivity during unpredictable stressors will predict poorer surrogate endpoint and clinical outcomes overall. Therapeutic 8 mg dose effects of doxazosin will be greater among alcoholics who display higher pre-treatment reactivity to unpredictable stressors.

AIM 4: Examine if the effects of doxazosin on clinical outcome measures are mediated by a reduction of stress-reactivity as measured by the NPU task. The aim is to identify whether reductions in stress-reactivity (NPU task) is the mechanism through which doxazosin has its effect on drinking behavior (clinical outcome).

PREDICTIONS: The direct effect of doxazosin (vs. placebo) following 8 weeks of therapeutic 8 mg dosing on clinical outcomes (e.g., continuous abstinence, drinking days/week, drinks/week) will be partially mediated by the indirect effect of doxazosin on surrogate endpoint of NPU stress reactivity at 4 weeks.

Official Title

Randomized Controlled Trial Targeting Noradrenergic Stress Mechanisms in Alcoholism With Doxazosin

Conditions

Alcoholism

Intervention / Treatment

- Drug: Doxazosin
- Other: Placebo

Other Study ID Numbers

- 2015-1009
- R01AA024388 (U.S. NIH Grant/Contract) (https://reporter.nih.gov/quickSearch/R01AA024388)
- A487400 (Other Identifier) (OTHER: UW Madison)

- L&S\PSYCHOLOGY\PSYCHOLOGY (Other Identifier) (OTHER: UW Madison)
- Protocol Version 11/7/2019 (Other Identifier) (OTHER: UW Madison)

Study Start (Actual)

2017-04-12

Primary Completion (Actual)

2020-03-13

Study Completion (Actual)

2020-03-13

Enrollment (Actual)

61

Study Type

Interventional

Phase

Phase 2

Resource links provided by the National Library of Medicine

MedlinePlus (https://medlineplus.gov/) related topics: Alcohol Use Disorder (AUD) (https://medlineplus.gov/alcoholusedisorderaud.html) Anxiety (https://medlineplus.gov/anxiety.html) Drug Safety (https://medlineplus.gov/drugsafety.html)

Drug Information (https://dailymed.nlm.nih.gov/dailymed/) available for: Doxazosin (https://dailymed.nlm.nih.gov/dailymed/search.cfm?labeltype=human&query=Doxazosin)

FDA Drug and Device Resources (https://clinicaltrials.gov/fda-links)

Contacts and Locations

This section provides contact details for people who can answer questions about joining this study, and information on where this study is taking place.

To learn more, please see the Contacts and Locations section in How to Read a Study Record (https://clinicaltrials.gov/study-basics/how-to-read-study-record#contacts-and-locations).

This study has 1 location

United States

Wisconsin Locations

Madison, Wisconsin, United States, 53706
University of Wisconsin

Participation Criteria

Researchers look for people who fit a certain description, called eligibility criteria. Some examples of these criteria are a person's general health condition or prior treatments.

For general information about clinical research, read Learn About Studies (https://clinicaltrials.gov/study-basics/learn-about-studies).

Eligibility Criteria

Description

INCLUSION CRITERIA:

- Diagnostic and Statistical Manual (DSM-5) diagnosis of Alcohol Use Disorder, Moderate-Severe
- Alcohol abstinent for 1 - 8 weeks
- Ages of 18 to 65

Exclusion criteria are divided into three broad categories of Medical, Psychiatric/Behavioral, and Medications/Therapies.

EXCLUSION CRITERIA: Medical

- Blood alcohol concentration above 0.00.
- Color blind.
- Heart rate >100 beats per minute after five minutes seated.
- Heart rate <55 beats per minute after five minutes seated.
- Systolic BP <100 after five minutes seated.
- Systolic BP drop >20mm Hg or diastolic BP drop >10mm Hg after two minutes standing.
- Dizziness, lightheadedness, unsteadiness or other problems (e.g, nausea, blurry vision) after two minutes standing.
- Uncorrected auditory/vision problems.
- Current treatment for chronic pain condition.
- Past or current coronary artery disease, cerebrovascular accident, congestive heart failure.
- Current chronic renal insufficiency, liver insufficiency or moderate hepatic impairment, pancreatitis, immunosuppressive therapy, or cancer with systemic effects or therapy.
- Benign positional vertigo, Meniere's disease, or narcolepsy.
- Previous allergic or adverse reaction to doxazosin or other alpha1 noradrenergic antagonist.
- Scheduled or reported plans for cataract surgery prior to study completion.
- Currently symptomatic of alcohol withdrawal [Clinical Institute Withdrawal Assessment for Alcohol, revised (CIWA-Ar) Score > 10, or positive for any 'visual, auditory or tactile disturbances,' or for 'orientation and clouding of sensorium']
- Discharged from inpatient treatment for Alcohol Use Disorder or alcohol detoxification within past 7 days.
- Currently medically unstable.
- Electrocardiogram (ECG) clinical over-read indicates concerns of cardiac function.

- Other self-reported acute or unstable illness that, in the opinion of the study team, would preclude a safe and reliable study participation

EXCLUSION CRITERIA: Female Participants Only

- Non-negative urine pregnancy test.
- Women of childbearing potential (see definition below) must agree to use one of the following forms of birth control until after study completion. Acceptable birth control is defined as the following methods of contraception: abstinence; hormonal contraceptives (e.g. combined oral contraceptives, patch, vaginal ring, injectables, and implants); intrauterine device (IUD) or intrauterine system (IUS); vasectomy of partner and tubal ligation; "single" barrier methods of contraception (e.g. male condom, female condom, cervical cap, diaphragm, contraceptive sponge) with use of spermicide; or "double barrier" method of contraception (e.g. male condom with diaphragm, male condom with cervical cap).
- Breastfeeding.

NOTE: Women of childbearing potential are females who have experienced menarche and do not meet the criteria for women not of childbearing potential. Women not of childbearing potential are females who are permanently sterile (e.g., hysterectomy, bilateral oophorectomy) or postmenopausal. Postmenopausal is defined as 12 consecutive months with no menses without an alternative medical cause.

EXCLUSION CRITERIA: Psychological/Behavioral

- Self-reported lifetime diagnosis of schizophrenia, schizoaffective disorder, psychotic disorder not otherwise specified, bipolar disorder (with manic episode), borderline personality disorder, or any neurocognitive disorder that may impair a reliable, safe participation.
- Current suicidal ideation.
- Current active substance use disorder other than alcohol or tobacco.

EXCLUSION CRITERIA: Medications/Therapies

- Currently prescribed or used within past week: doxazosin or other alpha1 noradrenergic antagonist (e.g., prazosin, terazosin).
- Currently prescribed or used within past week: substances with stimulant properties (e.g., d-amphetamine, methylphenidate, ephedra, pseudoephedrine).
- Currently prescribed or used within past week: Sildenafil (Viagra), tadalafil (Cialis), and vardenafil (Levitra).
- Currently prescribed or used within past week: beta-blockers (e.g., propanolol), alpha2 agonists (e.g., clonidine, guanfacine, dexmedetomidine), and serotonin and norepinephrine reuptake inhibitors (SNRI) (e.g., venlafaxine, duloxetine, atomoxetine,

viloxazine).

- Currently used daily or used within past week: alpha1 agonists (e.g., midodrine, metaraminol, oxymetazoline, phenylephrine).
- Currently used daily or used within past week: Benzodiazepines (e.g., diazepam, chlordiazepoxide, lorazepam, clonazepam, alprazolam), zolpidem (Ambien), zaleplon (Sonata), zopiclone (Imovane), eszopiclone (Lunesta), doxepin (Silenor).
- Currently prescribed and used daily or used within past 2 weeks: Trazodone.
- Currently prescribed or used within 2 weeks: Disulfiram (Antabuse).

Ages Eligible for Study

18 Years to 65 Years (Adult, Older Adult)

Sexes Eligible for Study

All

Accepts Healthy Volunteers

No

Study Plan

This section provides details of the study plan, including how the study is designed and what the study is measuring.

How is the study designed?

Design Details

Primary Purpose : Treatment
Allocation : Randomized
Interventional Model : Parallel Assignment
Masking : Triple (Participant, Investigator, Outcomes Assessor)

Arms and Interventions

Participant Group/Arm	Intervention/Treatment
Experimental: Doxazosin<br><br>Participants receive 8 weeks of doxazosin (8mg target dose).	Drug: Doxazosin<br><br>• Doxazosin
Placebo Comparator: Placebo<br><br>Participants will receive 8 weeks of matched placebo.	Other: Placebo<br><br>• Placebo

What is the study measuring?

Primary Outcome Measures

Outcome Measure	Measure Description	Time Frame

Startle Potentiation During Stress Reactivity Task	Startle potentiation is used to study anxiety and fear with No-shock, Predictable-shock, Unpredictable-shock (NPU) task; a common, well-validated laboratory stressor task. In the Predictable condition of the NPU task, shocks are 100 percent predictable and occur at a consistent, known time. In the Unpredictable condition of the NPU task, shocks are fully unpredictable. A higher score on startle potentiation means a higher stress reactivity response for the given condition.	4 weeks
Number of Participants Reporting Any Heavy Drinking Days	Timeline-followback (TLFB) was administered twice at 4 weeks and 8 weeks. Participants reported the number of drinks per day for each previous 30 day period. Any heavy drinking was scored "yes" if participant reported any days of heavy drinking (> 4/3 standard drinks for men/women) during the total 8 week assessment period; "no" if no heavy drinking was reported	8 weeks

Collaborators and Investigators

This is where you will find people and organizations involved with this study.

Sponsor

University of Wisconsin, Madison

Collaborators

- National Institute on Alcohol Abuse and Alcoholism (NIAAA)

Investigators

- Principal Investigator: John J Curtin, PhD, University of Wisconsin, Madison

Study Record Dates

These dates track the progress of study record and summary results submissions to ClinicalTrials.gov. Study records and reported results are reviewed by the National Library of Medicine (NLM) to make sure they meet specific quality control standards before being posted on the public website.

Study Registration Dates

First Submitted

2016-12-01

First Submitted that Met QC Criteria

2016-12-07

First Posted (Estimated)

2016-12-12

Results Reporting Dates

Results First Submitted

2021-02-23

Results First Posted with QC Comments

2021-03-17

Results First Submitted that Met QC Criteria

2021-03-16

Results First Posted

2021-03-18

Study Record Updates

Last Update Submitted that met QC Criteria

2021-03-16

Last Update Posted

2021-03-18

Last Verified

2021-03

More Information

Terms related to this study

Keywords Provided by University of Wisconsin, Madison

Alcoholism
Stress
Norepinephrine
Doxazosin
Anxiety
Startle Potentiation
Relapse
Adrenergic Antagonists
Surrogate Endpoint

Additional Relevant MeSH Terms

Alcohol-Related Disorders
Substance-Related Disorders
Chemically-Induced Disorders
Mental Disorders
Disease Attributes
Pathologic Processes
Pathological Conditions, Signs and Symptoms
Alcoholism
Anxiety Disorders
Recurrence
Prazosin
Quinazolines
Heterocyclic Compounds, 2-Ring
Heterocyclic Compounds, Fused-Ring
Heterocyclic Compounds
Substandard Drugs
Pharmaceutical Preparations
Doxazosin
Counterfeit Drugs

HHS Vulnerability Disclosure

Plan for Individual Participant Data (IPD)

Plan to Share Individual Participant Data (IPD)?

Yes

IPD Plan Description

Data will be made available online upon study completion at the Open Science Framework: https://osf.io

Drug and device information, study documents, and helpful links

Studies a U.S. FDA-Regulated Drug Product

Yes

Studies a U.S. FDA-Regulated Device Product

No

Product Manufactured in and Exported from the U.S.

No

Study Documents **Provided by University of Wisconsin, Madison**

- Study Protocol and Statistical Analysis Plan (https://cdn.clinicaltrials.gov/large-docs/93/NCT02989493/Prot_SAP_000.pdf) [PDF, 0.44MB, 2019-11-07]

Helpful Links Provided by University of Wisconsin, Madison

Dr. John Curtin's Addiction Research Center (http://arc.psych.wisc.edu)

ClinicalTrials.gov

NIH National Library of Medicine
National Center for Biotechnology Information

The U.S. government does not review or approve the safety and science of all studies listed on this website.

Read our full disclaimer (https://clinicaltrials.gov/about-site/disclaimer) for details.

Completed

Testing Doxazosin to Treat Stress Mechanisms in Alcoholism

ClinicalTrials.gov ID NCT02989493

Sponsor University of Wisconsin, Madison

Information provided by University of Wisconsin, Madison (Responsible Party)

Last Update Posted 2021-03-18

Results Posted Tab

Results Overview

Conditions

Alcoholism

Intervention/Treatment

Feedback

- Drug: Doxazosin
- Other: Placebo

Other Study ID Numbers

- 2015-1009
- R01AA024388 (U.S. NIH Grant/Contract) (https://reporter.nih.gov/quickSearch/R01AA024388)

Show 3 more study numbers

Study Design

Allocation: Randomized

Interventional Model: Parallel Assignment

Masking: Triple (Participant, Investigator, Outcomes Assessor)

Primary Purpose: Treatment

Results Point of Contact

Name/Title: John Curtin
Organization: University of Wisconsin
Phone: 608-262-0387
Email: jjcurtin@wisc.edu

Enrollment (Actual)

61

Study Type

Interventional

Study Record Dates

These dates track the progress of study record and summary results submissions to ClinicalTrials.gov. Study records and reported results are reviewed by the National Library of Medicine (NLM) to make sure they meet specific quality control standards before being posted on the public website.

Study Registration Dates

First Submitted

2016-12-01

First Posted (Estimated)

2016-12-12

Results Reporting Dates

Results First Submitted

2021-02-23

Results First Posted with QC Comments

2021-03-17

Results First Posted

2021-03-18

Study Record Updates

Last Update Posted

2021-03-18

Last Verified

2021-03

Participant Flow

Recruitment Details

Participants were enrolled via community advertisement and clinical referral.

Pre-assignment Details

61 participants were consented, passed medical screening, and were assigned to a drug group.

Arm/Group Title	Doxazosin	Placebo
Arm/Group Description	Participants receive 8 weeks of doxazosin (8mg target dose).<br>Doxazosin: Doxazosin	Participants will receive 8 weeks of matched placebo.<br>Placebo: Placebo

Period Title: **Overall Study**

Started	29	32
Completed	29	32
Not Completed	0	0

Baseline Characteristics

Arm/Group Title	Doxazosin	Placebo	Total
Arm/Group Description	Participants receive 8 weeks of doxazosin (8mg target dose).<br><br>Doxazosin: Doxazosin	Participants will receive 8 weeks of matched placebo.<br><br>Placebo: Placebo	Total of all reporting groups
Overall Number of Baseline Participants	29	32	61
Baseline Analysis Population Description	[Not Specified]		

Expand all / Collapse all

Age, Continuous [1]

Mean (Standard Deviation) | Unit of measure: years

Number Analyzed	29 participants	32 participants	61 participants
	45.6 (11.9)	39.7 (10.8)	42.5 (11.6)

[1] Measure Description: self report age

Sex: Female, Male

Measure Type: Count of Participants | Unit of measure: Participants

Number Analyzed	29 participants	32 participants	61 participants
Female	6 20.7%	9 28.1%	15 24.6%
Male	23 79.3%	23 71.9%	46 75.4%

Ethnicity (NIH/OMB)

Measure Type: Count of Participants | Unit of measure: Participants

Number Analyzed	29 participants		32 participants		61 participants	
Hispanic or Latino	1	3.4%	2	6.3%	3	4.9%
Not Hispanic or Latino	28	96.6%	30	93.8%	58	95.1%
Unknown or Not Reported	0	0.0%	0	0.0%	0	0.0%

Race (NIH/OMB)

Measure Type: Count of Participants | Unit of measure: Participants

Number Analyzed	29 participants		32 participants		61 participants	
American Indian or Alaska Native	0	0.0%	0	0.0%	0	0.0%
Asian	0	0.0%	0	0.0%	0	0.0%
Native Hawaiian or Other Pacific Islander	0	0.0%	0	0.0%	0	0.0%
Black or African American	2	6.9%	4	12.5%	6	9.8%
White	27	93.1%	27	84.4%	54	88.5%
More than one race	0	0.0%	1	3.1%	1	1.6%

Unknown or Not Reported	0 0.0%	0 0.0%	0 0.0%

Region of Enrollment [1]

Measure Type: Number | Unit of measure: participants

Number Analyzed	29 participants	32 participants	61 participants
United States	29	32	61

[1] Measure Description: All participants were enrolled in Madison, WI, USA

Outcome Measures

Expand all / Collapse all

1. Startle Potentiation During Stress Reactivity Task

Type: Primary | Time Frame: 4 weeks

Description	Startle potentiation is used to study anxiety and fear with No-shock, Predictable-shock, Unpredictable-shock (NPU) task; a common, well-validated laboratory stressor task. In the Predictable condition of the NPU task, shocks are 100 percent predictable and occur at a consistent, known time. In the Unpredictable condition of the NPU task, shocks are fully unpredictable. A higher score on startle potentiation means a higher stress reactivity response for the given condition.
Time Frame	4 weeks

Analysis Population Description	Although all 61 participants were able to provide TLFB data through the end of 8 weeks, the NPU task required that they attend a study visit at 4 weeks. 24 participants (10 in doxazosin, 13 in placebo) did not complete that study visit and so have data missing for this outcome measure. 1 (doxazosin) attended the visit but chose not to complete the NPU task. 2 participants (both placebo) completed NPU at this visit, but their NPU data was unusable for this analysis due to technical issues.	
Arm/Group Title	Doxazosin	Placebo
Arm/Group Description	Participants receive 8 weeks of doxazosin (8mg target dose).<br>Doxazosin: Doxazosin	Participants will receive 8 weeks of matched placebo.<br>Placebo: Placebo
Overall Number of Participants Analyzed	18	17
Unpredictable startle potentiation *Mean (Standard Deviation) | Unit of Measure: microvolts	22.25 (20.18)	20.76 (23.21)
Predictable startle potentiation *	26.22 (30.90)	22.21 (35.93)
* Mean (Standard Deviation) | Unit of Measure: microvolts		

2. Number of Participants Reporting Any Heavy Drinking Days

Type: Primary | Time Frame: 8 weeks

Description	Timeline-followback (TLFB) was administered twice at 4 weeks and 8 weeks. Participants reported the number of drinks per day for each previous 30 day period. Any heavy drinking was scored "yes" if participant reported any

	days of heavy drinking (> 4/3 standard drinks for men/women) during the total 8 week assessment period; "no" if no heavy drinking was reported	
Time Frame	8 weeks	
Analysis Population Description	[Not Specified]	
Arm/Group Title	Doxazosin	Placebo
Arm/Group Description	Participants receive 8 weeks of doxazosin (8mg target dose).<br>Doxazosin: Doxazosin	Participants will receive 8 weeks of matched placebo.<br>Placebo: Placebo
Overall Number of Participants Analyzed	29	32
Measure Type: Count of Participants | Unit of Measure: Participants	17 58.6%	21 65.6%

Adverse Events

Time Frame

8 weeks

Adverse Event Reporting Description

[Not Specified]

Arm/Group Title	Doxazosin	Placebo
Arm/Group Description	Participants receive 8 weeks of doxazosin (8mg target dose).<br>Doxazosin: Doxazosin	Participants will receive 8 weeks of matched placebo.<br>Placebo: Placebo

Expand

All-Cause Mortality

Arm/Group Title	Doxazosin	Placebo
	Affected / at Risk (%)	Affected / at Risk (%)
Total	0/29 (0.00%)	0/32 (0.00%)

Serious Adverse Events

Arm/Group Title	Doxazosin		Placebo	
	Affected / at Risk (%)	# Events	Affected / at Risk (%)	# Events
Total	0/29 (0.00%)		0/32 (0.00%)	

Other (Not Including Serious) Adverse Events

Frequency Threshold for Reporting Other Adverse Events	5%	
Arm/Group Title	Doxazosin	Placebo

Arm/Group Title	Doxazosin		Placebo	
Arm/Group Description	Participants receive 8 weeks of doxazosin (8mg target dose).<br>Doxazosin: Doxazosin		Participants will receive 8 weeks of matched placebo.<br>Placebo: Placebo	
	Affected / at Risk (%)	# Events	Affected / at Risk (%)	# Events
Total	24/29 (82.76%)		28/32 (87.50%)	
**Cardiac disorders**				
Cardiac arrhythmia †	8/29 (27.59%)	12	6/32 (18.75%)	7
Chest pains †	2/29 (6.90%)	5	0/32 (0.00%)	0
Edema †	3/29 (10.34%)	6	0/32 (0.00%)	0
**Ear and labyrinth disorders**				
Dizziness †	9/29 (31.03%)	17	7/32 (21.88%)	9
**Eye disorders**				
Blurry vision †	4/29 (13.79%)	4	0/32 (0.00%)	0
**Gastrointestinal disorders**				
Nausea or diarrhea †	9/29 (31.03%)	14	8/32 (25.00%)	9
**General disorders**				
Fatigue †	15/29 (51.72%)	38	17/32 (53.13%)	36

Arm/Group Title	Doxazosin		Placebo	
Arm/Group Description	Participants receive 8 weeks of doxazosin (8mg target dose).<br>Doxazosin: Doxazosin		Participants will receive 8 weeks of matched placebo.<br>Placebo: Placebo	
Rhinitis †	13/29 (44.83%)	29	5/32 (15.63%)	9
Headache †	9/29 (31.03%)	25	11/32 (34.38%)	17
Dry mouth †	10/29 (34.48%)	30	8/32 (25.00%)	15
General symptoms, nonspecific †	8/29 (27.59%)	9	6/32 (18.75%)	10
**Renal and urinary disorders**				
Polyurea †	2/29 (6.90%)	6	4/32 (12.50%)	8
**Reproductive system and breast disorders**				
Priapism † [1]	2/19 (10.53%)	2	0/20 (0.00%)	0
**Respiratory, thoracic and mediastinal disorders**				
Dyspnea †	4/29 (13.79%)	4	3/32 (9.38%)	5
**Vascular disorders**				
Orthostatic hypertension †	14/29 (48.28%)	24	7/32 (21.88%)	18

† Indicates events were collected by systematic assessment

Arm/Group Title	Doxazosin	Placebo
Arm/Group Description	Participants receive 8 weeks of doxazosin (8mg target dose).<br><br>Doxazosin: Doxazosin	Participants will receive 8 weeks of matched placebo.<br><br>Placebo: Placebo

Limitations and Caveats

[Not Specified]

Collaborators and Investigators

This is where you will find people and organizations involved with this study.

HHS Vulnerability Disclosure

Sponsor

University of Wisconsin, Madison

Collaborators

- National Institute on Alcohol Abuse and Alcoholism (NIAAA)

Investigators

- Principal Investigator: John J Curtin, PhD, University of Wisconsin, Madison

More Information

Record History

Certain Agreements

Principal Investigators ARE employed by the organization sponsoring the study.
