# Supplementary material for: Doxazosin for the treatment of mental health disorders: A scoping review
Source: PLOS Ment Health. 2025 Nov 19;2(11):e0000494. doi: 10.1371/journal.pmen.0000494 (PMC12798304; doi:10.1371/journal.pmen.0000494)
Supplement: Attachment — Submitted filename: Response to Reviewers .docx [file pmen.0000494.s005.docx]

**Response to Reviewers**

**Reviewer #1:**

1. The manuscript addresses an important clinical question, but the central research objective could be more explicitly stated in the Introduction.

**Response: Added clear objective to introduction.**

2. Organization is generally logical, though the dense results table would benefit from being split by diagnostic category for readability.

**Response: One table split up into 3 tables based on diagnostic categories for better readability.**

3. There is excessive repetition between the Results and Discussion sections; condensing the Results and deepening the analysis is advised.

**Response: Results section was condensed. Analysis in discussion was evaluated and improved.**

4. Mechanisms of action are well summarized, but a visual schematic would enhance reader understanding.

**Response: Visual schematic (original, not from another publication) added regarding chronic stress effect on brain and HPA.**

5. Differences between IR and XL formulations are mentioned but require clearer comparative analysis, particularly regarding tolerability.

**Response: Added comparative analysis in the discussion section on PTSD and nightmares regarding tolerability between IR and XL formulations.**

6. The review would be strengthened by a more detailed explanation of the heterogeneity in populations and its implications for generalizability.

**Response: Discussed more about heterogeneity in populations in discussion sections.**

7. References are comprehensive, but a follow-up on clinical trials marked as “unpublished” may reveal newer published data.

**Response: Reviewed unpublished clinical trials and did not find newer published data. New (8/2025) article about the ClonDoTrial does not have any results published yet so met exclusion criteria. Changed manuscript wording to emphasize that the two added clinical trials have no associated publications.**

8. Case reports use the term “remission” ambiguously—please clarify the criteria used for this designation.

**Response: Clarified that remission was complete resolution of symptoms.**

9. Language is generally professional, though occasionally leans into overinterpretation; be cautious when inferring causality.

**Response: Reviewed manuscript to remove overinterpretation and inferred causality.**

10. The authors assert that doxazosin may be more tolerable than prazosin but provide limited comparative data to support this.

**Response: Included tolerability data for prazosin vs doxazosin in discussion.**

**Reviewer #2:**

General comments:
The paper is adding information on the alternative therapy of using Doxazosin in the management of mental health disorders, which are increasingly a serious problem worldwide. Doxazosin has been reported in the treatment of various mental illnesses including PTSD and PTSD-related nightmares. However, the paper is addressing its role in other mental illnesses. However, following are some comments that need to be addressed.

The authors need to work on the grammar and the use of tenses throughout the paper.

**Response: Grammar and tenses were reviewed and edited.**

The authors need to clearly differentiate the noradrenergic system pathway in the brain and the sympathetic nervous system (SNS) is a division of the autonomic nervous system and a component of the peripheral nervous system, which is responsible for the body's "fight-or-flight" response. The derangement in the noradrenergic system pathways in the brain is responsible for development of mental disorders especially the depression, anxiety and many others. Therefore the authors need to clearly distinguish this. The noradrenergic system pathway has various receptors located in the brain and SNS periphery that may be targeted by drugs like alpha 1 (α1) antagonists such as doxazosin, prazosin etc., that may also cross the BBB to influence the brain and hence their use in mental illnesses.

**Response: Clarified and differentiated the noradrenergic system pathway in mental health disorders.**

1. Abstract
The authors should provide a background on the topic addressed in the paper. The aim of the review or research question addressed in the paper is not clear and it needs to be re-written. The statement in line 34-35, “--- included all types of human research, including interventional and observational clinical trials, case series/reports, and unpublished clinical trials”. How were the unpublished clinical trials retrieved, and this also falls in the gray literature? The study also seem not to have followed the “PICO (or PICOS) since a number of papers with different study designs were used in the review. Can the authors clarify this? Then, the papers that were not in PubMed, Embase, Cochrane, and PsycINFO were not considered and this possibly created the selection bias, how was this handled? Couldn’t the use of search engines also provide additional information on the subject? The authors should clearly state who exactly did the final screening of the papers to be included in the final review process. The conclusion in the abstract need to be paraphrased based on the key findings of the review paper.

**Response: Research question has been re-written. Access of unpublished clinical trials has been explained. Selection bias has been added to discussion. Discussion of not using PICOS was added to methods. Final screening of papers author was added. Abstract conclusion has been re-written and includes key findings paraphrased.**

2. Introduction
Line 57, the authors should include the role of the HPA (Hypothalamic-Pituitary-Adrenal) axis in the modulation of the body's response to stress which in turn may influence mental health. The aim of the review or research question addressed in the paper is not clear and it needs to be re-written (see above comment in the abstract). Specifically, which mental disorders where targeted by doxazosin and therefore used in the review?

**Response: Included statement about HPA axis in mental health. Re-wrote research question.**

3. Methods
The study also seem not to have followed the “PICO (or PICOS) since a number of papers with different study designs were used in the review. Can the authors clarify this? Then, the papers that were not in PubMed, Embase, Cochrane, and PsycINFO were not considered and this possibly created the selection bias, how was this handled? Couldn’t the use of search engines also provide additional information on the subject? The authors should clearly state who exactly did the final screening of the papers to be included in the final review process. The conclusion in the abstract need to be paraphrased based on the key findings of the review paper (see above comments in the abstract). The authors could also have used the PICO or PICOS in the eligibility criteria especially in the inclusion. The authors should also include a section on how included papers were retrieved, screened and who made the final screening of the papers that were used in the review.

**Response: PICO not being followed has an explanation added in methods. Selection bias was added and addressed in discussion. Final screening of papers was addressed in methods. Information was added in how papers were retrieved, screened, and final screening was done.**

4. Results
The authors should cite the table and figure in the text. Also, because of lack of use of PICO or PICOS in the study design, the authors couldn’t aggregate the findings. Suggestion, if possible; can the authors’ further aggregate the finding (outcomes) of those papers with similar study design?

**Response: Tables and figure cited in text. Studies aggregated by study design.**

5. Discussion
The authors should also highlight the key limitations of the review.

**Response: Key limitations added to discussion.**

5. Conclusion
The authors should highlight the key findings of the review with the title and objective if the review as guide.

**Response: Key finds highlighted in conclusion.**

**Reviewer #3:**

Strengths
Timely and relevant topic: Off-label use of doxazosin in psychiatry is underexplored, and this review compiles dispersed literature.

Comprehensive literature search: Databases (PubMed, Embase, Cochrane, PsycINFO) were systematically searched following PRISMA-ScR.

Clear categorization: The review differentiates between PTSD/nightmares, substance use disorders, and dual diagnoses.

Consideration of pharmacogenetics: Highlights genotype-specific response patterns in cocaine use disorder.

Clinical relevance: The paper identifies gaps in current practice and provides a foundation for future trials.

Major Weaknesses & Recommendations
Methodological Limitations

No risk-of-bias assessment was performed for included studies. While this is a scoping review, a brief discussion of study quality would strengthen the interpretation of findings.

**Response: Added discussion of study quality in discussion and also to each specific topic discussion.**

Many included studies are case reports or small open-label trials, yet this limitation is not sufficiently emphasized in the abstract and conclusion.

**Response: Added emphasis of limitations in abstract and conclusion.**

Presentation & Structure

Tables are detailed but dense. Condensed summary tables (e.g., separating RCTs from case reports) would improve readability.

**Response: Separated tables by diagnosis and added separation by study type.**

Figures such as a PRISMA flow diagram should be included to visualize study selection.

**Response: Figure 1 was added in the main manuscript.**

Interpretation of Findings

Conclusions could be more cautious regarding clinical recommendations, emphasizing the limited RCT evidence.

**Response: Changed conclusion recommendations to emphasize limited RCT evidence.**

The manuscript should explicitly discuss differences between doxazosin IR and XL formulations, as tolerability and dosing appear critical.

**Response: Added differences in formulations.**

References and Consistency

Some references are repeated (e.g., De Jong et al., 2010b is cited twice). Ensure reference style is consistent with PLOS guidelines.

**Response: Repeat reference removed. References changed to Vancouver style in line with PLOS guidelines.**

Language & Style

Minor grammatical and typographical issues should be revised for clarity and conciseness.

**Response: Grammar and tenses were reviewed and edited.**

The abstract could more clearly reflect the limited evidence and exploratory nature of the review.

**Response: Limited evidence and exploratory nature of review included.**
